# Holder-free single-port laparoscopic appendectomy as solo surgery and learning curve analysis of 100 cases

**DOI:** 10.1007/s13304-025-02203-3

**Published:** 2025-06-23

**Authors:** Haorun Lyu, Yuxi Li, Peng Guo, Shunlei Wang, Chuanlin Wang, Limin Guo, Lei Guo, Jiayang Liu, Weiqi Wang, Xiaoyu Fan, Zhiyong Li, Jie Yang

**Affiliations:** Department of Emergency Surgery, Peking University People’s Hospital, Beijing, 100044 China

**Keywords:** Appendectomy, Single-port laparoscopy, Solo surgery, Acute appendicitis

## Abstract

Acute appendicitis (AA) is a common condition that is typically treated with laparoscopic appendectomy (LA). Single-port laparoscopic appendectomy (SLA) offers potential benefits, such as reduced trauma and improved cosmesis. This study introduces a novel technique, Holder-Free Solo-SLA (HFSSLA), which is performed without an assistant or mechanical arm. A retrospective analysis of 100 consecutive HFSSLA patients performed by a single operator was conducted. The technique involved a single 2 cm umbilical incision and intra-abdominal operation using conventional laparoscopic equipment. The learning curve was assessed using CUSUM and MF-CUSUM analyses. According to the CUSUM and MF-CUSUM curves, inflection points occurred in the 36th and 26th patients, respectively. The mean operative duration was 55.58 min, which decreased to 50 min after the learning curve inflection point. Postoperative complications and cosmetic scores improved significantly after this point. This study demonstrated that as surgical experience increased, the efficiency and the safety of the operation improved. HFSSLA is a feasible technique for treating AA, with improved outcomes as surgeon familiarity increases. The technique's potential benefits should be weighed against its technical challenges and risks. Further research is needed to optimize the procedure and assess its applicability in various patient populations.

## Introduction

Acute appendicitis (AA) is one of the most common causes of lower abdominal pain that leads patients to the emergency department. The mortality rate for acute non-gangrenous AA is less than 0.1%, but it increases to 0.6% for gangrenous AA, and once perforated, the mortality rate increases to approximately 5% [1]. Laparoscopic appendectomy (LA) was first described by Semm in 1983 [2]. Current evidence suggests that LA is the most effective surgical treatment for AA [1]. The advantages of minimally invasive surgical techniques include ease of intraoperative exploration of the abdominal and pelvic cavities and wound distancing from the lesion, which in turn leads to a lower incidence of incisional infections, faster recovery, less pain, and better esthetics [3].

Single-port laparoscopic surgery is performed by reducing the trauma to the abdominal wall to achieve the goals of faster recovery, less pain and greater patient satisfaction. It also reduces the risk of wound infection due to a reduction in the number of abdominal wall wounds. Single-port laparoscopic appendectomy (SPLA/SLA) was first reported in 2003 [4]. Compared with conventional multi-port laparoscopic appendectomy (CLA), SLA lacks triangulation between instruments, and the entry of multiple instruments into the abdominal cavity through a single passageway can be more crowded, with instruments bumping into each other, making it difficult to maintain traction and counter-traction of the target tissue [5].

Solo-surgery refers to surgery performed by an operator without the need for an assistant. The concept of solo-laparoscopic surgery [6] was originally proposed to solve the problem of hand–eye incoordination of the operator during laparoscopic surgery using a special bracket to hold the lens, and the operator dominating the lens can effectively prevent the operator from attempting to understand the inappropriate operation, resulting in an unsatisfactory intraoperative field of view. This technique requires a mechanical device to fix the lens, which is limited by the equipment, and if it does not rely on the assistant to move the lens, the operator's field of view will be absolutely fixed when he does not have a free hand, which leads to the slow development of this technique. This research describes an innovative surgical approach for single-operator single-port laparoscopic appendectomy without reliance on mechanical arm assistance (Holder-Free Solo-SLA, HFSSLA, hereafter SSLA), which employs conventional laparoscopic equipment and instruments and eliminates the need for an assistant or a dedicated device to hold the lens in place by controlling the lens and the instrument responsible for retracting and fixing it simultaneously with the left hand, while the right hand carries out further maneuvers. In this study, the learning curve was plotted to analyze the process of mastery of the technique, while peri-operative and intraoperative data were retrospectively analyzed to assess the learning difficulty and clinical effectiveness of the technique.

## Methods

### Study population

The clinical data of 100 consecutive patients diagnosed with acute appendicitis based on laboratory tests and imaging studies and underwent SSLA at the Peking University People's  Hospital from February to June 2023 were retrospectively analyzed, and the surgeries were completed by the same operator without the use of exclusion criteria. The study was reviewed by the Ethics Committee of the XXX. All patient data were anonymized prior to analysis and consent was obtained. 

### Surgical technique

#### Surgical preparation

**Fig. 1:**
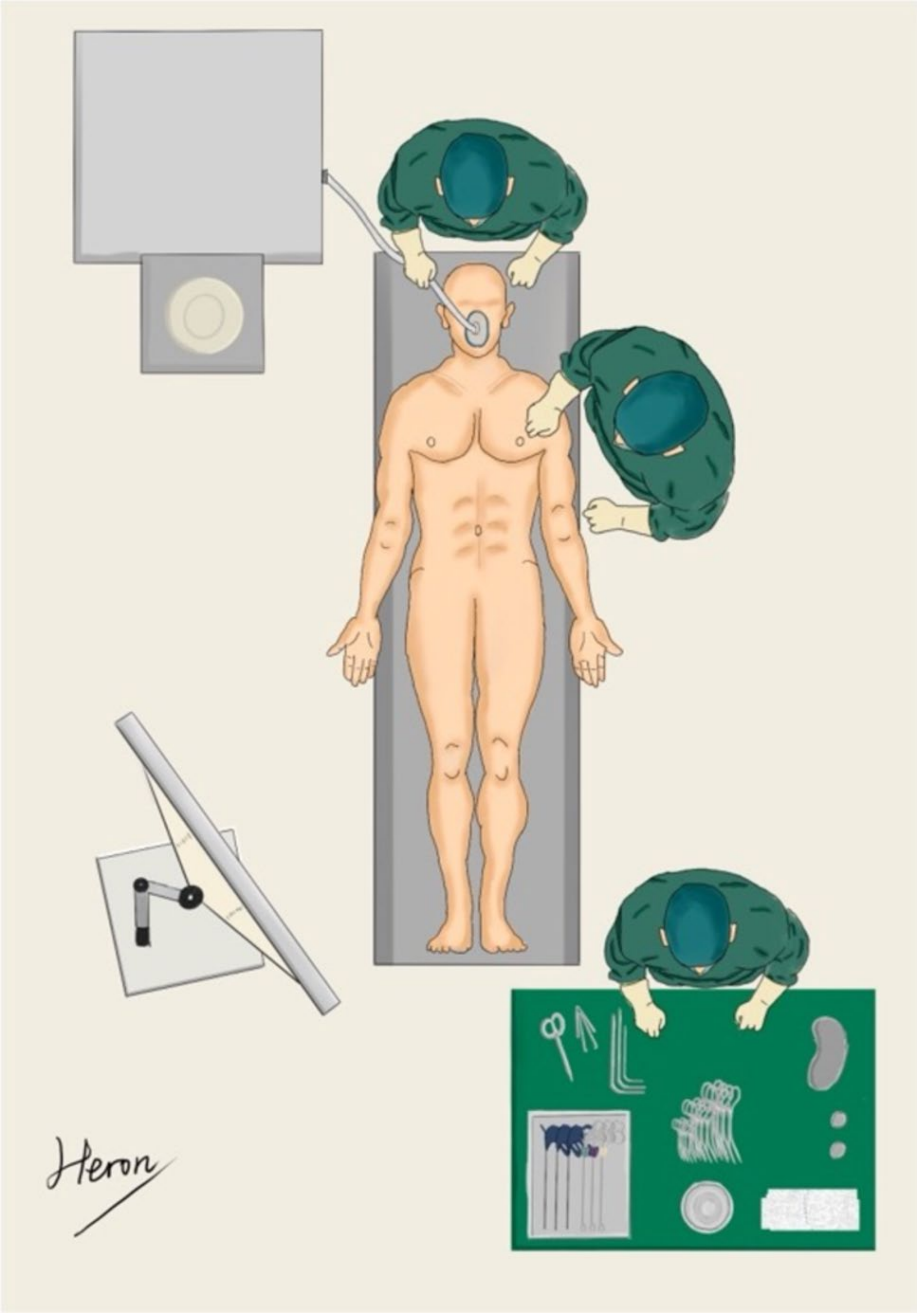
Layout of surgical equipment. The patient was placed in the supine position, the laparoscopic screen was located on the patient's right side near the caudal aspect, and the operator was on the patient's left side near the cephalic end

The procedure is similar to that for the CLA (Fig. 1): after successful anesthesia, the patient was placed in a lying position. The laparoscopic monitor was placed on the right side of the patient in a caudal position, and the operator was located on the left side of the patient. Laparoscopic lens, light source, suction, and energy instruments were attached, and the author was accustomed to using an ultrasonic scalpel, along with a 10 mm laparoscopic lens and noninvasive laparoscopic instruments. Only a conventional gastric grasping forceps and a suction were usually required.

#### Channel establishment

**Fig. 2:**
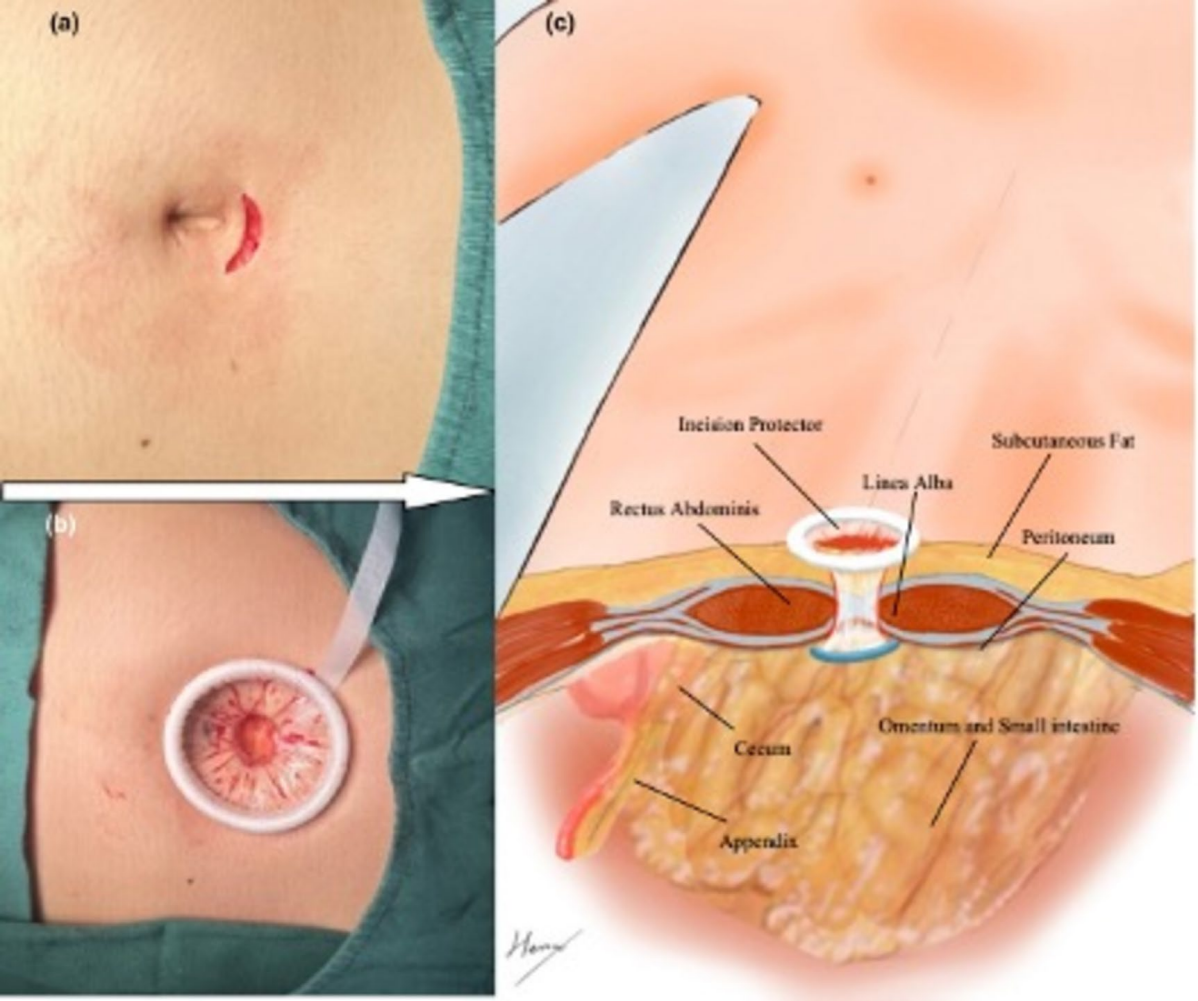
Photographs and schematic diagram of the surgical access establishment process. **a** Appearance of the incision, with the arrow direction on the side of the patient's head; **b** Effect after placing the incision protector, with the arrow direction on the side of the patient's head; **c** Schematic diagram of anatomic relationships

A 2 cm curved incision was made at the upper edge of the umbilical ring, and forceps were placed in both hands to bluntly separate subcutaneous fat and rectus abdominis muscle until the white line was reached (Fig. 2). The white line was lifted longitudinally clamped and cut through the tip. After the clamp tip was inserted, the clamp was opened to expand the channel, and the peritoneum was lifted. After confirming that the intra-abdominal organs were not damaged, the peritoneum was incised in the same way, and the channel was enlarged to a caliber comparable to that of the skin incision. The incision protection sleeve was inserted, and then a single-port laparoscopic operating platform (Aerospace Cadet) was fastened and connected to the pneumoperitoneum with a pressure of 12 mmHg to complete the channel establishment.

#### Intra-abdominal operation

The patient's position was adjusted to the head-low-right-high position. The left hand was used to hold the camera, the right hand was used to hold the forceps for appendiceal positioning and posture adjustment, and the right-hand instruments were replaced with the suction device for cleanup if there was combined contamination. Where adhesions need to be loosened, the right-hand grasping forceps are handed over to the left hand to maintain the position and tension of the appendix, while the right hand holds the suction device for blunt separation or the ultrasonic scalpel for sharp dissection. Once the dissection is satisfactory, the grasping forceps are lifted to the appendiceal root or mid-section and handed over to the left hand. Tension is applied and maintained to the appendiceal root by a finger-to-palm maneuver with the left hand, and the right hand treats the appendiceal mesenteric vessels with an ultrasonic scalpel to the appendiceal root to the point of nudity. A vascular clip may be used to close the appendiceal artery as appropriate to prevent intraoperative hemorrhage, and the right-hand instruments are replaced with vascular clips and clamped to close the appendiceal root lumen 2 proximally and 1 distally before severing the appendix and finally removing it through the incision. In patients with significant root swelling or perforation at the root, in whom it is difficult for vascular clamps to securely and reliably close the root, the root of the appendix is resected along with a portion of the cecum using linear cutting closure, and the severed end is reinforced with a series of barbed sutures. Specific tips for each step of the procedure are summarized in the Appendix.

### Follow-up strategy

Patients were followed up at 1, 7 and 42 days postoperatively for postoperative complications, postoperative pain, effect of surgery on body image and confidence level, wound satisfaction and overall satisfaction ratings. Intraoperative and postoperative complications were defined as follows [5]: conversion to open surgery, bleeding, organ damage, wound infection, postoperative bowel obstruction, intra-abdominal abscess formation, and appendiceal stump inflammation. Postoperative pain was evaluated using the VAS score, where a score of 0 represents no pain at all, and a score of 10 points represents pain that is completely intolerable. BIQ (Body Image Questionnaire, BIQ) [7] evaluates the effect of surgery on body image and surgical incision satisfaction and is divided into 3 parts—body image score, cosmetic score and self-confidence score—with a total of 10 questions, of which the body image score is 5 questions, and each question is 1–4 points; the lower the score is, the higher the evaluation, and the maximum evaluation is 5 points. The beauty score for the 3 questions ranged from 1 to 7/7/10 points, and the higher the score was, the greater the evaluation. The self-confidence score consists of 2 questions, with 1 ~ 10 points for each question; the higher the score is, the greater the evaluation.

### Indicators and evaluation criteria

Patient demographics included sex, age (years), height (m), weight (kg), BMI (kg/m^2^), and comorbidities, while comorbidities were recorded using the age-corrected Charlson Comorbidity Index (CCI) [8]. CCI includes a weighted scoring of a range of chronic diseases and health conditions, with each condition assigned a different score. Additionally, the CCI takes into account patient's age, with points added for each decade of life starting from the age of 50, with an increment of 1 point for every additional 10 years. Disease-related indicators included the length of the interval from disease onset to consultation, the history of previous abdominal surgery, and indicators of preoperative infections, including leukocyte count (10^9^/L), C-reactive protein concentration (mg/L), and prolactin concentration (ng/mL). We measured the maximum diameter of the appendix using the patient's preoperative CT and differentiated whether the patient's appendicitis was complex according to imaging criteria, the specific signs of which included peri-appendiceal fat stranding, abscess formation, extraluminal air, pneum-appendicosis, appendicolith, peri-appendiceal fluid/ascites and ileus [9]. Surgical-related indicators included the length of the operation, intraoperative hemorrhage, postoperative pathology, incidence of surgical complications, and presence or absence of drainage tube placement, and follow-up indicators included the VAS score and BIQ score on postoperative days 1, 3, 7, and 42. 

### Learning curve analysis

Cumulative sum analysis (CUSUM) has been widely used to assess learning curves [10]. A rising trend indicates that the practitioner is in the learning stage of the technique, a reaching inflection point indicates that the learning stage is over and the practitioner has mastered the technique, and a subsequent downward trend indicates that the practitioner has mastered the technique.

In this study, surgical time was used for observation, that is, the total time from scalpel contact with the skin to the completion of suturing. The data were plotted by calculating the difference between the observed value and the mean value for each sample. The CUSUM was calculated using the following formula:

$${\mathrm{CUSUM}} = \sum\nolimits_{{{\mathrm{i}} = 1}}^{{\mathrm{n}}} {\left( {{\mathrm{xi}} - {\mathrm{x}}} \right)}$$

$\mathrm{xi}$ is the duration of surgery for each patient, $\mathrm{x}$ is the mean duration of surgery for all patients, and $\mathrm{n}$ is the serial number of the patient, which ranges from 1 to 100 in this study. A scatterplot was plotted with the CUSUM as the vertical coordinate and the serial number of the surgery as the horizontal coordinate and fitted to obtain the learning curve equation, which was solved to find the inflection point of this curve by solving for f'(x) = 0 after derivatives were taken. The patients were divided into two groups according to the presence of inflection points, and the preoperative, intraoperative, and postoperative data of the patients were compared to exclude significant differences between the two groups.

To conduct multifactor CUSUM (MF-CUSUM) analysis, we selected the length of surgery, intraoperative bleeding, and postoperative complications as the outcome indicators, and the quantitative values of these three assessment indicators for each patient were a1, a2, and a3, respectively. The quantitative values of the assessment indicators were defined as a = Xi-X0, where Xi is the actual situation of a particular surgery and X0 is the target expectation. Xi = 1 indicates that the criterion has not been met, and Xi = 0 indicates that the criterion has been met, such as surgery time exceeding expectations or the occurrence of complications. In this case, the target surgery duration was set to 60 min, and 34 out of 100 patients exceeded this standard, X0 = 0.34. The target value of bleeding was set to 10 mL, and a total of 19 cases exceeded this target, X0 = 0.19. The actual number of complications was 11, X0 = 0.11. The quantitative value of surgical competence for each patient was defined as S = a1 + a2 + a3. After the completion of each case, the scores were summed sequentially and then plotted by the following formula:

$${\mathrm{MF}} - {\mathrm{CUSUM}} = \sum\nolimits_{{{\mathrm{i}} = 1}}^{{\mathrm{n}}} {{\mathrm{Si}}}$$

### Statistical analysis

Statistical analysis was performed using R software (version 4.2.2, R Foundation for Statistical Computing, Vienna, Austria). Normally distributed continuous variables are expressed as mean and standard deviation. Categorical variables are expressed as frequencies. Differences in characteristics between groups were analyzed by chi-square test for categorical variables and t test for continuous variables. *P* values less than 0.05 were considered to indicate statistical significance.

## Results

### Patient baseline data and surgery-related indicators

A total of 100 cases of SSLA were performed by the operator between February and June 2023. According to the postoperative pathologic findings, 33 patients had simple appendicitis, 19 had purulent appendicitis, 24 had gangrenous appendicitis, and 24 had appendiceal cellulitis. The mean age of the patients was 40.84 ± 16.85 years, the mean BMI was 23.92 ± 3.73 kg/m^2^, and the mean duration of disease onset was 46.37 ± 37.54 h. The mean outer diameter of the appendix on CT was 11.42 ± 3.58 cm, and 65 of 100 patients met the diagnostic criteria for complicated appendicitis.

### Plotting the learning curve of SSLA with CUSUM

**Fig. 3:**
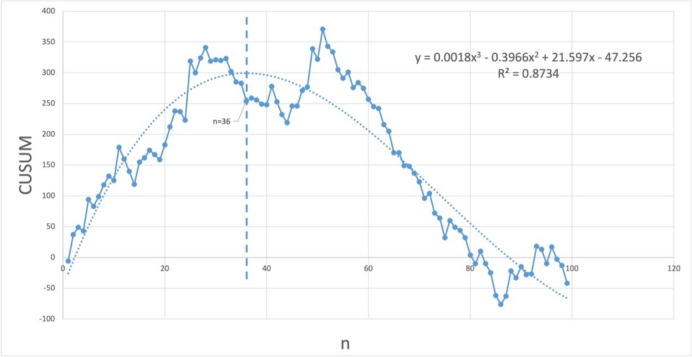
The Learning Curve of SSLA with CUSUM

Based on the CUSUM calculations, the learning curve equation obtained from the fitting is $\mathrm{CUSUM}=0.0018{\mathrm{x}}^{3} - 0.3966{\mathrm{x}}^{2} + 21.752\text{x }- 60.411{\mathrm{R}}^{2}=0.87$, as shown in Fig. 3. The curve has a typical upward and downward trend, indicating the familiarization and mastery phases of the technology. Derivation of the equation f*'* (CUSUM)=0.0054x^2^-0.7932x+21.752, f*'* (CUSUM = 0), and $\mathrm{x}=36$ shows that the inflection point occurs in the 36th surgical case.

In Table 1, we divided the patients into before and after groups using the calculated inflection point (*n* = 36) as the demarcation point and tested the differences in the preoperative, intraoperative and postoperative indices between the two groups. Duration of surgery, intraoperative bleeding, and postoperative cosmetic scores of the two groups were significantly different (*p* = 0.014, 0.006, 0.027), and the patients in the After group were better than those in the Before group. Moreover, there was a difference in the complication status of the patients in the two groups (*p* = 0.046), and the patients in the After group had more complications. Other than these indicators, there was no significant difference between the two groups of patients (*p* > 0.05).

**Table 1:** Demographic data and surgical outcomes of patients who underwent surgery for SSLA (*n* = 100)

	Total, *n* = 100	Before, *n* = 36	After, *n* = 64	t/χ^2^	*p*
Preoperative information					
Age (years)	40.84 ± 16.85	38.75 ± 16.28	42.02 ± 17.18	t = -0.94	0.348
Sex					
Male n(%)	48 (48.0%)	18 (50.0%)	30 (46.9%)	χ^2^ = 1.00	0.317
Female n(%)	52 (52.0%)	18 (50.0%)	34 (53.1%)	χ^2^ = 1.00	0.317
BMI (kg/m^2^)	23.92 ± 3.73	24.02 ± 3.92	23.86 ± 3.65	t = 0.20	0.845
Duration of onset (hours)	46.37 ± 37.54	42.56 ± 37.63	48.52 ± 37.62	t = -0.76	0.450
Outer diameter (cm)	11.42 ± 3.58	10.83 ± 2.93	12.25 ± 4.24	t = -1.84	0.075
Complex appendicitis n(%)	65 (65.0%)	25 (69.4%)	40 (62.5%)	χ^2^ = 0.07	0.798
Laboratory experiment					
WBC (10^9^/L)	12.44 ± 4.52	12.39 ± 5.01	12.47 ± 4.26	t = -0.07	0.941
CRP (mg/L)	40.44 ± 55.63	43.98 ± 53.27	38.45 ± 57.23	t = 0.49	0.629
PCT (ng/mL)	1.93 ± 6.31	3.59 ± 9.55	1 ± 3.06	t = 1.58	0.122
Charlson Score	1.38 ± 1.82	0.89 ± 1.3	1.55 ± 1.94	t = -2.02	0.046
Surgical Data					
S-Time (minutes)	55.58 ± 24.04	63.86 ± 25.82	50.92 ± 21.83	t = 2.54	0.014
Bleeding (mL)	11.03 ± 11.97	16.58 ± 17.55	7.91 ± 5.15	t = 2.90	0.006
Complications n(%)	11 (11.0%)	1 (2.8%)	10 (15.6%)	χ^2^ = 0.17	0.684
Drain n(%)	19 (19.0%)	12 (33.3%)	7 (10.9%)	χ^2^ = 0.56	0.453
Abdominal surgery n(%)	10 (10.0%)	3 (8.3%)	7 (10.9%)	χ^2^ = 0.41	0.522
Pathologic type					
Simple appendicitis n(%)	33 (33.0%)	11 (30.6%)	22 (34.4%)	χ^2^ = 6.79	0.659
Septic appendicitis n(%)	19 (19.0%)	11 (30.6%)	8 (12.5%)		
Gangrenous appendicitis n(%)	24 (24.0%)	6 (9.4%)	18 (28.1%)		
Appendiceal cellulitis n(%)	24 (24.0%)	8 (22.2%)	16 (25.0%)		
Follow-up outcome					
Body image score (5 ~ 20)	5.41 ± 0.87	5.44 ± 1.16	5.39 ± 0.66	t = 0.26	0.799
Cosmetic score (3 ~ 24)	21.51 ± 3.33	20.36 ± 4.36	22.16 ± 2.38	t = -2.29	0.027
Day 3 VAS (1 ~ 10)	3.91 ± 1.93	3.64 ± 2.09	4.06 ± 1.84	t = -1.02	0.313

### Plotting the learning curve of the SSLA with the multifactor CUSUM

**Fig. 4:**
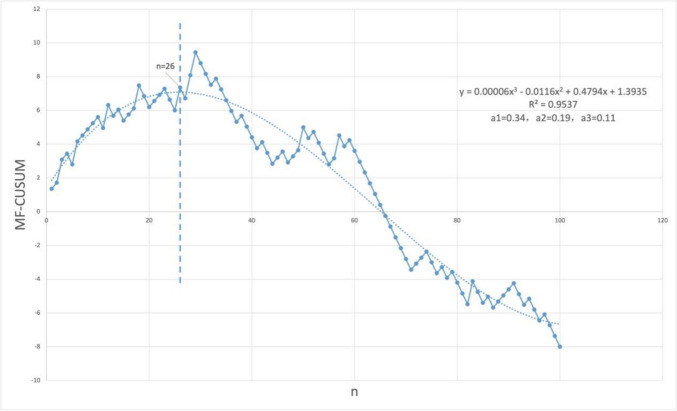
The Learning Curve of the SSLA with the Multifactor CUSUM

On the basis of the analysis of surgical duration, we proceeded to introduce intraoperative bleeding and the occurrence of postoperative complications for RF-CUSUM curve plotting (Fig. 4). The equation obtained by fitting the curve is $\mathrm{MF}-\mathrm{CUSUM}=0.00006{\mathrm{x}}^{3} - 0.0116{\mathrm{x}}^{2} + 0.4794\mathrm{x}+1.3935 {\mathrm{R}}^{2}=0.95$. This curve also has a clear upward and downward trend and clear inflection points. The MF-CUSUM curve equation was better fitted than the CUSUM curve equation (R^2^ = 0.95). Again, the derivation of this equation, f*'* (MF-CUSUM) = 0.00018x^2^-0.00232x+0.4794 f*'* (CUSUM) = 0, and $\mathrm{x}=26$, yields the inflection point at the 26th surgical case.

## Discussion

SLA offers benefits over traditional laparoscopic surgery (CLA), including fewer abdominal wall incisions, reduced incision pain for patients, and a lower incidence of trocar-related complications. However, SLA also faces several technical challenges, such as instrument handles obstructing each other, limited operating range, insufficient tension during retraction and exposure, prolonged operating time, and compromised vision [11]. The European Association for Endoscopic Surgery (EAES) Consensus Statement on Single-Incision Endoscopic Surgery [12] notes that although SLA has a longer learning curve, it provides better cosmetic outcomes, shorter hospital stays, and faster return to work. The consensus also summarized the results of 11 studies comparing SLA and CLA (*n* = 1437) and revealed that the SLA group had significantly better postoperative cosmetic scores than did the CLA group, with comparable mean operative times (52 min in the SLA group and 49 min in the CLA group), and that there was no difference in the rate of adverse events between the two groups, which confirms the safety and effectiveness of the SLA procedure.

In this study, SSLA, a new and improved technique of SLA, had a mean operative duration of 55.58 min, which did not increase the time consumed by SLA. After crossing the inflection point to master the technique, the average procedure duration was reduced to 50 min. In terms of long-term complications, SLAs have a greater likelihood of incisional hernia than CLAs due to the use of 1 relatively large channel. SSLA further improves upon SLA by enabling the use of an entry route between rectus muscles and reducing intraoperative bleeding, postoperative pain, and muscle function limitations, thus accelerating patient recovery. Incisional hernia was not returned in all 100 cases in this study.

This technique eliminates the dependence on fixation equipment and assistants while actively increasing the difficulty of the procedure to some extent, and because it is an innovative technique, its safety and efficacy need to be tested before it can be generalized. The innovations of this technique are as follows:

1. An original incision and access establishment method, which saves money, improves efficiency, reduces injury, and enhances esthetics;
2. An original maneuver of holding the camera and forceps in the left hand simultaneously and the ultrasonic scalpel or vascular clamp in the right hand, which is named the Heron technique here;
3. An original approach to the camera is to achieve the simultaneous insertion of two 10 mm instruments into the 2 cm channel without interfering with each other.

Kim et al. proposed a single-person, single-port laparoscopic appendectomy technique [13], but this technique requires the use of a special skin retraction device (Lone Star hook) and a special mechanical arm for laparoscopic fixation. Hardware requirements and costs have limited the promotion of this technique. The technique in this paper is free from the limitations of external equipment and can be performed by a single person using only conventional laparoscopic surgical instruments, which significantly relieves the current pressure of tight medical resources in emergency medicine.

In acute appendicitis, due to the possibility of gangrene and perforation, a delay in the operation may lead to an increase in the extent of contamination. The time from disease onset to surgery directly affects the therapeutic effect and patient prognosis. The application of this technology eliminates the need to configure and convene surgical assistants, which means reducing manpower consumption and expenditure while greatly improving treatment efficiency and providing treatment to more patients under the same conditions.

With the increasing use of single-port laparoscopy, a wide variety of assistive devices, such as rigid curved instruments, articulated bendable instruments, and even camera-holding robots, have been introduced, but none of them are used in this technology. The only difference between this technology and traditional laparoscopy surgery is the single-port laparoscopy-specific operating platform, which means that all centers capable of carrying out laparoscopic surgeries are able to carry out SSLA without increasing additional costs. Moreover, from a health economics point of view, personnel costs account for 50% of surgical expenditures ^[[14]]^, and solo-surgery can reduce surgical costs by 10–15% due to the reduced need for surgical personnel.

The potential risks of this technique are the same as those of conventional laparoscopic surgery, including intraoperative finding that the appendix is difficult to resect laparoscopically and the difficulty of relying on a single person to perform intraoperative surgery when there is accidental injury to vital organs or hemorrhage and a need to quickly complete the repair or hemostasis operation. The main risk of this technique lies in the fact that if an emergency situation arises during an operation that requires urgent treatment, it is difficult to respond quickly to the lack of manpower available on the table. Therefore, at the initial stage, other physicians must be present to participate in the operation at any time and at the same time be fully prepared to increase the number of auxiliary ports at any time to switch back to traditional laparoscopy or even transit to open surgery. At the same time, patient's condition and general situation should be fully evaluated before the operation, and the difficulty and the risk of the operation should be taken into account to determine whether this technique is suitable. For surgeons who have not yet mastered the single-port technique, it is recommended that patients with low surgical difficulty, such as those with relatively mild inflammation and a low body mass index, be selected at the beginning of the procedure to increase the success rate of the operation and to ensure surgical safety. As the technique matures, it can be applied to more difficult and complex appendectomies. Operators who assess the likelihood that a procedure cannot be performed by a single person should not choose to utilize this technique when manpower support is not available in a timely manner, but it can be considered when a team is needed to respond to multiple emergency procedures at the same time and can support each other in a timely manner, which can dramatically increase the efficiency of the procedure.

The technique used in this study was completely new. Our results showed that the operating time decreased and intraoperative bleeding decreased with increasing surgical experience, which indicated that the efficiency and safety of the operation improved. At the same time, the establishment of the operating procedure requires an increase in the number of cases, while appendectomy, as the first intra-abdominal procedure learned and mastered by the operator (a low seniority attending physician) in this study, who had no prior experience in laparoscopic surgery, would require fewer cases for an experienced surgeon to become proficient in this technique.

In this study, surgical outcomes were evaluated based on metrics, such as operative time, intraoperative bleeding, complication rates, and postoperative cosmetic scores. Based on the CUSUM and MF-CUSUM learning curves, surgeons experienced a steeper learning curve in the early stages and became progressively more skillful as the number of surgeries increased. This suggests that although SSLA technology is difficult to learn, surgeons are able to gradually master and optimize the surgical process through continued practice and experience. This improvement not only increases the operational efficiency of the operating room but also may reduce the patient's anesthesia exposure time, thus reducing anesthesia-related risks. The reduction in intraoperative bleeding further confirms the improvement in surgical precision, which may be related to the surgeon's better understanding of anatomic structures and improved manipulation skills.

## Conclusion

Through the analysis of 100 SSLA surgery patients, this study assessed the relationship between surgeons’ familiarity with the technology and surgical outcomes. The results show that as surgical experience increases, the operation time decreases, and intraoperative blood loss decreases, indicating improved surgical efficiency and safety. Future research should further explore the effects of SSLA technology in different population groups and optimize the surgical process to improve the accessibility and quality of medical services, thereby increasing the overall health of society.

## Data Availability

The data that support the findings of this study are available from the corresponding author upon reasonable request.

## Appendix: SSLA operating techniques and essentials (Heron technique)

1. Choice of instruments—Duckbill: Single-port laparoscopy permits the formation of a small angle due to the loss of the triangular relationship between the instruments (chopstick effect). In most cases, only one laparoscopic instrument is allowed to operate in the abdominal cavity. We recommend the use of duckbill forceps (gastric grasping forceps) as the angle of the tip can be rotated and hooked, and other actions can increase flexibility, partially compensating for the shortcomings of single-instrument operations.
2. Tension Maintenance—Spaghetti: In gastrointestinal surgery, maintaining proper tissue tension is critical to the success of the procedure, and in single-port laparoscopy without an assistant, it is not possible to provide and maintain tissue tension by contralateral retraction. The grasping forceps can be rotated to tighten the tissue to provide tension without moving the position of the instrument and then manipulated, similar to rolling spaghetti with a fork.
3. Instruments in and out of the channel—8: Since the maximum diameter of the surgical channel is only 2 cm or even smaller, when it is necessary to pass two 10 mm laparoscopic instruments (lenses and vascular clamps) at the same time, the lens is withdrawn to the level of the incision, the clamp is inserted, the incision is straightened, and the space for inserting the lens is squeezed through twisting and drilling.
4. Solo operation—Chord C: The right hand holds the duckbill forceps and grasps the appendix for retraction and exposure. After the position is satisfactory, the forceps are handed over to the left hand, which holds the lens and grasping forceps at the same time. The grasping forceps were used to enter the right channel, the appendix was pulled to the opposite side (left side of the screen), and the right operating space was expanded (a). Alternatively, the grasping forceps are inserted from the upper channel while the left hand releases the lens and turns to hold the light source line, using the lens's own gravity to maintain a stable field of view, pulling the appendix toward the ipsilateral side (the right side of the screen), and inserting the suction device or ultrasonic scalpel using the right channel to perform the operation (b). The left-hand position for this step is similar to that of guitar chord C.
5. For appendiceal closure-vacancy, at least 3 Hem-o-loks needed to be clamped during surgery. After clamping 1, the hemolock clamp was removed to fix the position of the other instruments, and the clamp could be directly inserted into the abdominal cavity through the original channel to avoid repetition of the lens. For cases where the root is perforated or too swollen to be clamped shut, it is necessary to use a barbed suture, which is technically difficult with only one hand, and it is recommended to add auxiliary holes if necessary.
6. Removal of the specimen—Evacuate: After the appendix is cut off, the grasping forceps are kept on the severed end of the appendix, and the platform is opened to remove the specimen directly; this process usually takes only 5 s, which is very rapid, and contamination of the appendix in the peritoneal cavity, as well as the incision, is avoided. However, in the acute inflammatory period, the appendix may be obviously swollen, and the diameter of the channel is only 1.5 ~ 2 cm. It is recommended to pay attention to the abdominal organs directly below the incision (usually the omentum) to determine whether they are contaminated by drops of appendiceal exudate or even fecal matter in the cavity and to clean them in time.
